# High Background Congenital Microcephaly in Rural Guatemala: Implications for Neonatal Congenital Zika Virus Infection Screening

**DOI:** 10.9745/GHSP-D-17-00116

**Published:** 2017-12-28

**Authors:** Anne-Marie Rick, Gretchen Domek, Maureen Cunningham, Daniel Olson, Molly M Lamb, Andrea Jimenez-Zambrano, Gretchen Heinrichs, Stephen Berman, Edwin J Asturias

**Affiliations:** a Department of Pediatrics, University of Colorado School of Medicine, Aurora, CO, USA.; b Center for Global Health, Colorado School of Public Health, Aurora, CO, USA.; c Department of Epidemiology, Colorado School of Public Health, Aurora, CO, USA.; d Pediatric Infectious Diseases, University of Colorado School of Medicine, Aurora, CO, USA.; e Department of Obstetrics and Gynecology, University of Colorado School of Medicine, Aurora, CO, USA.

## Abstract

**Background:**

Congenital microcephaly is the result of a disturbance in early brain development and can have multiple etiologies. Establishing background prevalence of microcephaly in Zika virus (ZIKV)-affected areas is important for improving identification of ZIKV-affected newborns. However, to date, there is limited consistent guidance for the accurate identification of microcephaly in infants of unknown gestational age, a common concern in low- and middle-income countries.

**Methods:**

Occipital frontal head circumference (OFC) obtained from infants (0–13 days) of unknown gestational age at enrollment in a pregnancy registry in rural Guatemala from August 2014 to March 2016 were retrospectively reviewed. Trained community health nurses recorded anthropometry in an online database. In April 2015, ZIKV was identified in this population. Gestational age was approximated in 2 ways: presumed term and estimated using z-score of zero for height on modified Fenton growth curves. After which, z-scores for OFC and weight were obtained. Microcephaly and microcephaly background prevalence were estimated using 7 established microcephaly case definitions from national and international organizations and 3 proposed definitions using Fenton growth curves. Independent associations with microcephaly and OFC, including relationship with date of birth, were assessed with prevalence ratios and linear regression.

**Results:**

For 296 infants, the mean OFC was 33.1 cm (range, 29.5 to 37 cm) and the mean OFC z-score was −0.68. Depending on case definition, 13 to 125 infants were classified as having microcephaly (background prevalence 439 to 4,223 per 10,000 live births), and 1 to 9 infants were classified as having severe microcephaly (<−3 standard deviation [SD]) (34 to 304 per 10,000 live births). Five (1.7%) infants met all the microcephaly case definitions. Weight ≤−1 SD (prevalence rate [PR], 3.77; 95% confidence interval [CI]: 1.6 to 8.8; *P*=.002) and small for gestational age (PR, 4.68; 95% CI, 1.8 to 12.3; *P*=.002) were associated with microcephaly. Date of birth was not associated with OFC z-score or OFC after adjusting for gestational age and gender.

**Conclusions:**

Estimated background microcephaly is high in rural Guatemala compared with reported rates in Latin America prior to ZIKV epidemic, which has important implications for neonatal screening programs for congenital ZIKV infection. Fenton growth curves offer a standardized approach to the identification of microcephaly in infants of unknown gestational age.

## Abstract (precis)

A variety of microcephaly case definitions detect high background prevalence in rural Guatemala, which complicates congenital Zika screening efforts. In addition, gestational age is needed for most screening tools but is usually unknown in low-resource settings. Fenton growth curves, originally designed for use in preterm infants, offer a standardized approach to adjust for unknown gestational age and may improve screening efforts.

## INTRODUCTION

Congenital microcephaly is the result of a disturbance in early brain development leading to an abnormally small head circumference and structural abnormalities of the brain, and can have multiple etiologies.^1^ Prior to the Zika virus (ZIKV) epidemic, the estimated prevalence of microcephaly in Latin America was 3.30 per 10,000 live births; in Brazil it was 1.98 per 10,000 live births.^2^^,^^3^ As of 2015, Brazil has reported a microcephaly rate more than 20 times higher than thepre-epidemic rate, which has been attributed to congenital ZIKV infection.^3^^,^^4^ However, the use of more conservative and stringent diagnostic criteria reflected in the case definitions, combined with an under-recognition of microcephaly prior to the ZIKV epidemic, may have contributed to the low background prevalence estimates of this condition.^5^ Prior to the ZIKV epidemic, the Latin American Collaborative Study of Congenital Malformations (ECLAMC) defined microcephaly as a head circumference measuring greater than 3 standard deviations (SD) below the mean growth curve, adjusted for age and gender.^6^ In contrast, current case definitions use a cut-off of greater than 2 SD below the mean, or below the third percentile of the growth curve, in addition to identifying structural brain abnormalities.^1^ Hence, the proportion of microcephaly attributed to ZIKV may be overestimated, which may have widespread implications for congenital ZIKV infection screening programs.

The proportion of microcephaly attributed to ZIKV may be overestimated and, therefore, may have widespread implications for congenital ZIKV infection screening programs.

Measurement of the occipital frontal head circumference (OFC) is the screening tool used to identify infants with microcephaly who may suffer or not from a structural brain abnormality. However, interpretation of OFC is dependent upon gestational age, gender, and race, which makes provision of a universal screening cut-off for microcephaly challenging. Furthermore, in the setting of the ZIKV epidemic, it is important that the microcephaly case definition leads to the identification of the greatest number of congenital microcephaly cases while limiting false positives to avoid unnecessary medical evaluations—specialist visits, neuroimaging, laboratory—and the associated financial costs and emotional stress. This has resulted in changing microcephaly case definitions throughout this ZIKV epidemic, using different methods and estimates.

The development of a consistent and accurate case definition is further complicated by the large number of infants born in low- and middle-income countries (LMICs) with unknown gestational age. In Guatemala, approximately 25% of women do not receive appropriate antenatal care, and only 65% of births are attended by a skilled birth attendant.^7^ Gestational age has been integral to case definitions of microcephaly used before and during the early ZIKV epidemic. However, it was not until August 2016 that World Health Organization (WHO) published the first recommendations for defining microcephaly in infants of unknown gestational age.^1^ It is important to note that these guidelines have several important limitations, including a tendency toward a high false positive rate, and do not address suspected premature infants of unknown gestational age. As a result, there is a pressing need to develop a more robust approach to identifying infants of unknown gestational age at risk for congenital ZIKV infection.

The development of a consistent and accurate case definition is further complicated by the large number of infants born in low- and middle-income countries with unknown gestational age.

Therefore, we sought to: (1) estimate the background prevalence of microcephaly in a neonatal population of unknown gestational age born primarily before the ZIKV epidemic in a rural area of Guatemala, using various case definitions of microcephaly as used in Latin America during the ZIKV epidemic; and (2) explore the applicability of new case definitions for microcephaly among our local population, which could serve as a better screening tool for microcephaly when gestational age is unknown.

## METHODS

### Population

A dataset collected as part of a quality improvement project of the Creciando Sanos community health program was retrospectively reviewed to examine OFC measurements obtained from infants (0–13 days) from August 1, 2014, to March 31, 2016. This longitudinal child growth and development program is operated by the Fundacion para la Salud Integral de los Guatemaltecos (FUNSALUD) and sponsored by the Fundacion Jose Fernando Bolanos and Agroamerica in the coastal lowlands, known as the southwest Trifinio region, located at the intersection of the departments of San Marcos, Quetzaltenango, and Retalhuleu, in rural Guatemala. Through the program, children 0 to 3 years are monitored by regularly scheduled home visits using health screenings and development assessments with trained community health nurses (CHNs). Although serologic evidence of ZIKV transmission was first identified in this area in April 2015, the first clinical cases were not reported by the Ministry of Health until November 2015.^8^^,^^9^

### Anthropometric Measurements

Anthropometric measurements were taken by CHNs who first measured body length, weight, and head circumference of newborns during their program enrollment visit, and then recorded the measurements in an online database. Body length was measured to the nearest 0.1 cm using a portable Seca measuring board (Seca 210, Chino, California, USA) for infants. Weight was recorded to the nearest 0.1 kg using a Salter Brecknell hanging scale (Fairmont, Minnesota, USA). The CHNs were instructed to use flexible tape measures to measure an infant's head circumference from the most prominent part of the forehead around to the widest part of the back of the head, and to measure at least 2 times, recording the largest number to the nearest 0.1 cm.

### Estimated Gestational Age

Determining gestational age by ultrasound or last menstrual period was not possible for all infants. Therefore, gestational age was estimated using 2 methods. First, all infants were assumed as having reached full term (≥37 weeks gestational age). This is a reasonable assumption as the majority of infants were home births, did not receive clinical interventions, and were all still living at time of enrollment. However, it is likely that at least some of these infants were actually preterm or late preterm—34 to 37 weeks gestational age—births. Second, all infants were given an estimated gestational age by centering their length at a z-score of zero on gender-adjusted Fenton growth curves. Fenton growth curves provide postnatal anthropometric growth standards for preterm infants derived from large population-based studies of infants born in developed countries.^10^^,^^11^ Once gestational age was estimated, percentiles and z-scores for OFC and weight could then be obtained on gender-adjusted Fenton growth curves.

### Microcephaly Case Definitions

A total of 7 case definitions for microcephaly with widespread use in Latin America during the ZIKV epidemic were identified through literature review (Table 1). Two case definitions from the Brazil Ministry of Health (MOH) were used, the first (MOH 1) during the early ZIKV epidemic—from approximately November 8 to December 8, 2015^12^—and the second (MOH 2) was a revised definition employed until approximately March 13, 2016.^13^ Two case definitions from the Pan American Health Organization (PAHO) were employed, the first (PAHO 1) was issued through an epidemiologic alert on December 1, 2015,^4^ and the second (PAHO 2) was a revised definition released in early 2016.^14^ The World Health Organization (WHO) issued new recommendations and guidelines (WHO 1–3) released in August 2016.^1^ While these most recent guidelines from WHO recommend that InterGrowth-21 curves be used as a reference standard in infants of known gestational age, for suspected term infants of unknown gestational age, the recommendation is to use WHO growth curves. We also defined 3 new case definitions based on estimated gestational age on the Fenton growth curves adjusted for gender for microcephaly in infants of unknown gestational age using cut-offs of <-2 SD (Fenton 1), below the third percentile (Fenton 2), and <-3 SD (Fenton 3)—the latter identifies severe microcephaly.

**TABLE 1.** Established and Proposed Microcephaly Case Definitions

Origin of Case Definition	Microcephaly Case Definition
Brazil MOH 1	Term: OFC ≤33.0 cm for all infants Preterm: OFC ≤3rd percentile Fenton GC adjusted for GA and gender
Brazil MOH 2	Term: OFC ≤32.0 cm for all infants Preterm: OFC ≤3rd percentile Fenton GC adjusted for GA and gender
PAHO 1	Term: OFC <−2 SD WHO GC for males (<31.9 cm) and females (<31.5 cm) Preterm: OFC <−2 SD Fenton GC adjusted for GA and gender
PAHO 2	Term: OFC <3^rd^ percentile WHO GC for males (<32.0 cm) and females (31.6 cm) Preterm: OFC <3^rd^ percentile Fenton GC adjusted for GA and gender
WHO 1	Unknown GA, suspected term: OFC <−2 SD WHO GC 0–6 days: males: <31.9 cm; females: <31.5 cm 7–13 days: males: <32.7 cm; females: <32.2 cm
WHO 2	Unknown GA, suspected term: OFC <3rd percentile WHO GC 0–6 days: males: <32.0 cm; females: <31.6 cm 7–13 days: males: <32.8 cm; females: <32.4 cm
WHO 3	Unknown GA, suspected term: OFC <−3 SD WHO GC^a^ 0–6 days: males: <30.7 cm; females: <30.3 cm 7–13 days: males: <31.5 cm; females: <31.1 cm
Fenton 1	All infants: <−2 SD Fenton GC adjusted for gender and estimated GA
Fenton 2	All infants: <3rd percentile Fenton GC adjusted for gender and estimated GA
Fenton 3	All infants: <−3 SD Fenton GC adjusted for gender and estimated GA^a^

Abbreviations: GA, gestational age; GC, growth curve; MOH, Ministry of Health; OFC, occipital frontal head circumference; PAHO, Pan American Health Organization; SD, standard deviation; WHO, World Health Organization.

a <-3 SD defines severe microcephaly.

A total of 7 case definitions for microcephaly were in widespread use in Latin America during the ZIKV epidemic.

### Identification of Microcephaly and Estimated Background Prevalence

The case definitions were then applied to our dataset to identify suspected cases of microcephaly in this population and estimate microcephaly background prevalence prior to the ZIKV epidemic. When the case definition required a gestational age, the estimated gestational age was derived from the Fenton growth curves. When the case definition did not require a gestational age, all infants were assumed full term (≥37 weeks gestational age). The percent agreement of identified suspected cases was then assessed by the established Brazil MOH, PAHO, and WHO case definitions and our proposed Fenton growth curve case definitions.

### Statistical Analysis

Using our proposed Fenton 2 case definition, associations of independent variables with microcephaly were explored with prevalence ratios on univariate analysis including weight ≥1 SD below the mean and small for gestational age. As some of the infants were born after identification of local ZIKV transmission, potential impact of ZIKV on microcephaly was explored in several ways. First, prevalence ratios were estimated for birth date as a continuous variable and for infants born before and after May 1, 2015—allowing for first local serologic evidence of ZIKV in April 2015—and before and after December 1, 2015—allowing for an approximate full-term gestation after onset of regional ZIKV epidemic and first clinical reports of ZIKV infection—using microcephaly as a binary outcome. Finally, in order to assess if there were changes in OFC over time, regression coefficients for birth date as a continuous variable were estimated using measured OFC and OFC z-score as continuous outcomes, first as a univariate analysis and then controlling for gender and estimated gestational age.

## RESULTS

### Anthropometric Measurements and Estimated Gestational Age

**FIGURE 1:**
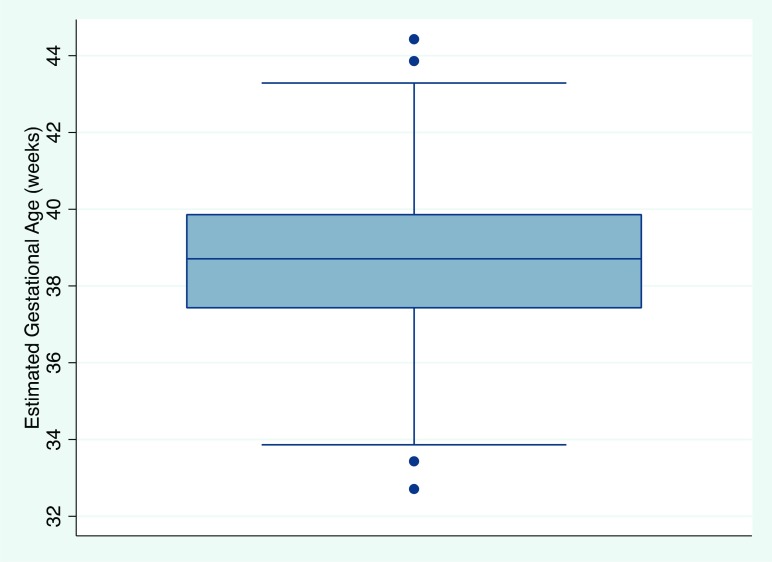
Box Plot With Whiskers of Estimated Gestational Age^a^ Note: Median gestational age is 38.7 weeks, and the interquartile range is 37.4 to 39.9 weeks. ^a^ Gestational age was estimated by centering an infant's height at a z-score of zero on gender-adjusted Fenton growth curves.

**FIGURE 2:**
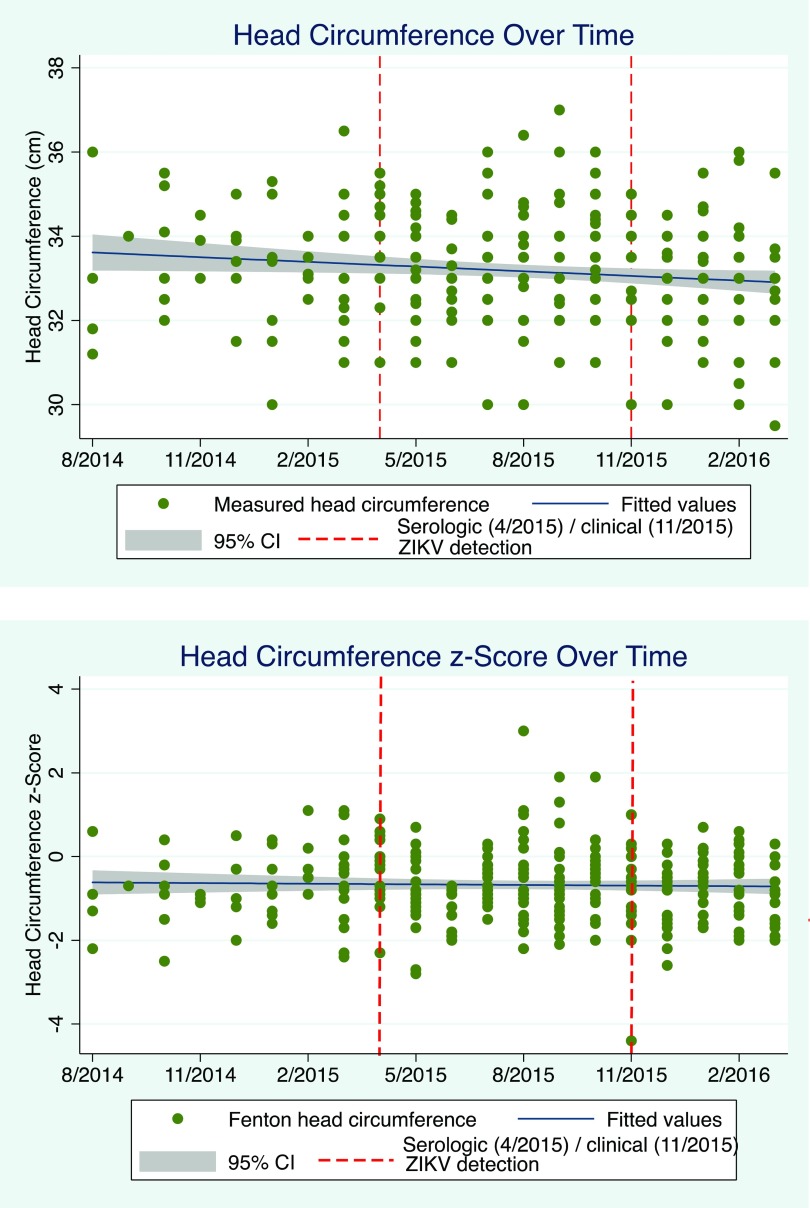
Measured Head Circumference and Z-Score for All Infants by Month of Birth

A total of 296 infants, ages 0 to 13 days old, were identified from the pregnancy registry with 1 exclusion due to erroneous length entry (Table 2). About a quarter (65; 22%) of the infants were born prior to May 1, 2015, while almost three-quarters (213; 72%) were born prior to December 1, 2015. Data on most (257; 87%) of the infants were collected in their first week of life: the mean OFC was 33.5 cm (range, 30 cm to 37 cm) for males and 32.9 cm (range, 29.5 cm to 36.4 cm) for females; the mean length was 50.0 cm (range, 43 cm to 55 cm) for males and 49.2 cm (range, 44 cm to 54 cm) for females, and the mean weight was 3.2 kg (range, 1.8 kg to 4.3 kg) for males and 3.1 kg (range, 2.2 kg to 4.5 kg) for females. The median estimated gestational age, based on the Fenton growth curves, was 38 weeks and 5 days (range, 32 weeks 5 days to 44 weeks 3 days) (Figure 1); the mean OFC z-score was −0.68 (95% confidence interval [CI], −0.78 to −0.58) (Figure 2); and the mean weight z-score was −0.12 (95% CI, −0.21 to −0.04).

**TABLE 2.** Characteristics of Neonates Born Between August 1, 2014, and March 31, 2016, in Rural Guatemala (N=296)

Characteristic	No. (%)
Gender	
Male	143 (48.3)
Female	153 (51.7)
Birth year	
2014	20 (6.8)
2015	214 (72.3)
2016	62 (20.9)
Age, days	
0–6	257 (86.8)
7–13	39 (13.2)
Weight, kg	
1.5 to <2.0	2 (0.7)
2.0 to <2.5	8 (2.7)
2.5 to <3.0	78 (26.4)
≥3.0	208 (70.3)
Length, cm	
40 to <45	4 (1.4)
45 to <50	139 (47.0)
≥50	153 (51.7)
OFC, cm	
≤30	7 (2.4)
>30 to ≤31	17 (5.7)
>31 to ≤32	48 (16.2)
>32 to ≤33	91 (30.7)
>33	133 (44.9)

Abbreviation: OFC, occipital-frontal head circumference.

### Identification of Microcephaly and Estimated Background Prevalence

The Brazil MOH 1 case definition identified the highest number of suspected cases of microcephaly, with 125 infants meeting the case definition, giving an estimated background rate of microcephaly of 4,223 cases per 10,000 live births (Table 3). The Brazil MOH 2 case definition identified the second highest number of suspected cases—48 with an estimated background rate of 1,622 cases per 10,000 live births—although the number was substantially lower than with the MOH 1 definition. The WHO 1 and 2 definitions, which reflected the current recommendations for infants of unknown gestational age, identified 36 and 43 infants, respectively, giving an estimated background prevalence between 1,216 and 1,453 cases per 10,000 live births. The PAHO 1 and 2 definitions identified 15 and 20 infants, respectively, giving an estimated background prevalence of between 507 and 676 cases per 10,000 live births. The proposed definitions of Fenton growth curves <−2 SD (Fenton 1) and less than third percentile (Fenton 2) identified 13 and 20 infants, respectively, giving an estimated background prevalence between 439 and 676 cases per 10,000 live births. The WHO 3 definition (<−3 SD on WHO growth curves) identified 9 infants with severe microcephaly, while the proposed Fenton 3 definition for severe microcephaly (<−3 SD on Fenton growth curves) identified 1 infant.

**TABLE 3.** Estimated Microcephaly Cases and Microcephaly Background Prevalence Using Established and Proposed Microcephaly Case Definitions in Neonates Born Between August 1, 2014, to March 31, 2016, in Rural Guatemala

Origin of Case Definition	Microcephaly No. (%)	Microcephaly Background Prevalence per 10,000 Live Births
Brazil MOH 1	125 (42.2)^a^	4,223
Brazil MOH 2	48 (16.2)^a^	1,622
WHO 2	43 (14.5)^b^	1,453
WHO 1	36 (12.2)^b^	1,216
PAHO 2	20 (6.8)^a^	676
Fenton 2	20 (6.8)^a^	676
PAHO 1	15 (5.1)^a^	507
Fenton 1	13 (4.4)^a^	439
WHO 3	9 (3.0)^b^	304
Fenton 3	1 (0.3)^a^	34

Abbreviations: MOH, Ministry of Health; PAHO, Pan American Health Organization; WHO, World Health Organization.

a Based on infant's estimated GA using z-score of zero for length on Fenton growth curve adjusted for gender. Term if estimated GA ≥37 weeks; preterm if estimated GA <37 weeks.

b Assumes term (≥37 weeks) GA for all infants.

Estimated background prevalence of microcephaly determined by the 10 studied case definitions ranged substantially— from 34 to 4,223 per 10,000 live births.

Thirteen (65%) infants identified by the Fenton growth curve less than third percentile definition (Fenton 2) and 6 (46%) infants identified by Fenton growth curve <−2 SD definition (Fenton 1) were also identified by the WHO growth curve less than third percentile definition (WHO 2) and WHO <−2 SD definition (WHO 1), respectively (Table 4). Overall, 5 (1.7%) infants met all case definitions for microcephaly (proposed and established) and 1 infant met the 2 definitions for severe microcephaly.

**TABLE 4.** Percent Agreement Between Established Microcephaly Case Definitions and Proposed Fenton Growth Curve Definitions

	Fenton GC <−2 SD (n=13)	Fenton GC <3rd Percentile (n=20)	Fenton GC <−3 SD (n=1)
	No. (%)	No. (%)	No. (%)
Brazil MOH 1	13 (100.0)	20 (100.0)	1 (100.0)
Brazil MOH 2	11 (84.6)	18 (90.0)	1 (100.0)
PAHO 1	5 (38.5)	11 (55.0)	1 (100.0)
PAHO 2	6 (46.2)	12 (60.0)	1 (100.0)
WHO 1	6 (46.2)	12 (60.0)	1 (100.0)
WHO 2	7 (53.8)	13 (65.0)	1 (100.0)
WHO 3	2 (15.4)	2 (10.0)	1 (100.0)
Identified on all case definitions	5 (38.5)	11 (55.0)	1 (100.0)

Abbreviations: GC, growth curve; MOH, Ministry of Health; PAHO, Pan American Health Organization; SD, standard deviation; WHO, World Health Organization.

### Factors Associated With Microcephaly

Weight ≤-1 SD (prevalence rate [PR], 3.77; 95% CI, 1.6 to 8.8; *P*=.002) and small for gestational age (PR, 4.68; 95% CI, 1.8 to 12.3; *P*=.002) were associated with microcephaly. Microcephaly was not associated with birth before or after May 1, 2015 (around when the first serologic Zika exposure was identified locally) (PR, 0.65; 95% CI, 0.3 to 2.6; *P*=.37), before or after December 1, 2015 (around when the first clinical Zika case was identified nationally) (PR, 0.64; 95% CI, 0.2 to 1.9; *P*=0.41), or birthdate (PR, 0.998; 95% CI, 0.995 to 1.001; *P*=0.25).

Measured OFC was found to be associated with birthdate (β −0.001; 95% CI, −0.002 to −0.0002; *P*=.02) on univariate analysis, but after adjusting for gender and estimated gestational age this association was no longer significant (β −0.0006, 95% CI, -0.002 to 0.0003; *P*=.19) (Figure 2). The OFC z-score was not associated with birthdate (β −0.0002; 95% CI, −0.0009 to 0.0006; *P*=.63) (Figure 2).

## DISCUSSION

Regardless of the case definition used, the estimated background congenital microcephaly of 34 to 4,233 per 10,000 live births in this rural community prior to and during Guatemala's early ZIKV epidemic was significantly higher than the overall background rate of 3.30 per 10,000 live births reported in Latin America before the ZIKV outbreak.^2^ Based on our analysis, using the current WHO congenital ZIKV screening guidelines would give a high false-positive rate and result in high numbers of referrals for diagnostic evaluation, creating significant ramifications at the individual, family, and community levels. These results raise important issues relevant to this community and other communities within LMICs affected by the ZIKV epidemic, including the need to further investigate the causality of high background rates of microcephaly and to determine the best screening methods and guidelines to be applied in areas where the gestational age of infants is often unknown. The substantial difference we identified in estimated background rates is likely the result of changing case definitions of microcephaly before and during the ZIKV epidemic as well as additional factors not accounted for in this analysis. Prior to the ZIKV epidemic, Brazil (and most of the world) defined microcephaly as <−3 SD, although some regions and hospitals did use alternative definitions.^2^^,^^3^ Subsequent to the ZIKV epidemic, less restrictive definitions were applied to improve the identification of suspected cases of congenital ZIKV infection. This was clearly illustrated in Brazil by Victora et al., where the sensitivity for definitions used during the ZIKV epidemic were between 80% and 92%, compared with 57% of the standard <-3 SD OFC definition.^15^

Using the current congenital ZIKV screening guidelines would give high false-positive rate and result in high numbers of referrals for diagnostic evaluation, creating significant ramifications at the individual, family, and community levels.

Underreporting of microcephaly prior to the ZIKV epidemic may also be contributing to these differences. A large retrospective review of head circumference in infants born prior to the ZIKV epidemic in Northeast Brazil (n=16,208) found that microcephaly rates were significantly higher than nationally reported rates over a similar time frame.^5^ Even when using a conservative cut-off of <-3 SD on Fenton growth curves adjusted for age and gender, their findings give an estimated rate of 3.7 cases per 10,000 live births—more than double the national rate reported in Brazil prior to ZIKV epidemic. It is reasonable to speculate that underreporting also occurred in other countries in Latin America where large proportions of infants are delivered outside of hospitals and where measurement of OFC is not obligatory.

The higher burden of microcephaly identified in our population may also reflect other population-specific factors, such as prenatal malnutrition, toxins, genetics, or other unrecognized congenital infections, like cytomegalovirus, that result in unique anthropometric characteristics at birth (smaller OFC, shorter length).^16^ Additionally, the level of microcephaly in our population may also be affected by the accuracy of anthropometric measurements taken by community health workers in the field. While accurate birth weights are often not known, especially for home deliveries, weight measurements can be successfully obtained shortly after birth at the community level.^17^ Accurate head circumference measurements, however, may be harder to obtain in the community setting. If the measurement is not taken around the widest possible circumference of the head, then the measurement may provide a false result. The Creciando Sanos program took several steps to try to minimize mistakes. The program employs auxiliary or professional CHNs who were trained to perform anthropometric measurements. Furthermore, the CHNs were required to measure the head circumference of each child at least 2 times and to record the largest number.

The level of microcephaly in our population may also be affected by the accuracy of anthropometric measurements taken by community health workers in the field.

Our findings have important implications for congenital ZIKV infection screening programs. Currently, once an infant with microcephaly is identified, additional screening procedures—such as physical and neurological evaluations, laboratory testing, and, often, neuroimaging—are recommended to confirm a congenital ZIKV infection.^1^ However, if a population has a high background prevalence of microcephaly, using a measurement that indicates microcephaly alone as a criterion for ordering more invasive and expensive screening will lead to overutilization of scarce resources and to increased emotional and financial burdens experienced by families. For example, after Brazilian state-level medical teams investigated the laboratory and neuroimaging results of more than 1,500 infants with suspected congenital ZIKV infection, more than half of the suspected cases were determined to be unlikely to be infected and, therefore, their results were discarded.^18^ Assuming the cost of neuroimaging in Brazil is comparable to the cost in rural Guatemala, where a head ultrasound is approximately US$20 and an MRI is $250, this means neuroimaging costs of between $15,000 and $187,500 may have been spent on suspected cases that were all ultimately discarded.

Exploring alternative microcephaly definitions for screening, particularly for infants of unknown gestational age, may be one way to significantly improve specificity while maintaining sensitivity and provide an alternative to the current screening practices for infants of known gestational age. Until the recent ZIKV epidemic, there has been limited discussion on how to approach microcephaly screening for infants of unknown gestational age. The application of a case definition that optimizes sensitivity and specificity without requiring a gestational age could help improve screening for ZIKV-affected infants. Although originally designed for use in preterm infants, the Fenton growth curves offer a standardized approach to addressing and adjusting for unknown gestational age. The use of the sensitive WHO growth curves for presumed full-term infants can result in a high false-positive rate, which was recognized as a limitation by WHO itself.^1^ This appears to be consistent with the findings in our dataset, where the WHO growth curves estimated a microcephaly background prevalence between 1,216 and 1,453 per 10,000 live births in our population. This overestimation may be due in part to an unknown percentage of infants being late-preterm births. In our experience with this Guatemalan community, the WHO growth curves would significantly overestimate the number of infants with congenital microcephaly and lead to an excessive and unnecessary referral pattern overburdening community health, material, and financial resources.

Approximately 46% to 65% of suspected cases on Fenton growth curves were also identified by the WHO definitions. Therefore, Fenton growth curves may offer an opportunity to capture infants of unknown gestational age—who are of the greatest concern for pathologic congenital microcephaly—while at the same time reducing false-positive rates. An additional advantage is that this method allows for identification of infants with asymmetric growth restriction—that is, disproportionately small heads and weight compared to length. Identification of disproportionate weight and OFC may prove useful as lower birth weight is associated with confirmed and probable congenital ZIKV cases compared with non-cases.^18^ However, the utility of using the Fenton growth curves as we did for identification of infants with microcephaly who have symmetric growth restriction—or proportionately small length, head circumference, and weight—is limited because of centering on a length z-score of zero.

Fenton growth curves may offer an opportunity to capture infants of unknown gestational age—who are of the greatest concern for pathologic congenital microcephaly—while at the same time reducing false-positive rates.

Despite this, the benefit of using the Fenton growth curves is that it accommodates infants whose anthropometric data are collected beyond the immediate delivery period. Although WHO recommends assessment of head circumference at 24 hours of life, many of the infants born in rural communities are not given an anthropometric assessment within this period. Therefore, if an infant of unknown gestational age is instead assessed during their second or third week of life, it becomes unclear which WHO growth curve is most appropriate to accurately assess for microcephaly as these growth curves are only available at weekly intervals. For example, an 8-day-old male infant with an OFC of 33 cm would be classified as greater than the third percentile if using the WHO 1-week cut-off of 32.9 cm but less than the third percentile if using the WHO 2-week cut-off of 33.7 cm. Meanwhile, the Fenton growth curves have standardized estimates for anthropometric data at daily intervals through 50 weeks gestational age. This means that using an infant's length at the time of first assessment to estimate a gestational age—which would also account for postnatal age—a more individualized assessment of the infant's anthropometric data can be obtained beyond the immediate delivery period.^10^

### Limitations

Several limitations need to be considered when using Fenton growth curves in the proposed manner. First, these growth curves were derived primarily from large dataset analysis of infants born primarily in developed countries. It has been well established that children in developing countries often have unique anthropometry compared to children of developed countries. Thus, the Fenton growth curves may not fully reflect the anthropometry of infants from LMICs. It may be more appropriate to center length on a z-score other than zero to accurately estimate gestational age in certain LMICs. However, since we primarily use the Fenton growth curves to identify disproportional head circumference compared to length, this bias may not significantly impact our results. We did consider employing a similar approach with InterGrowth-21 growth curves, which provide fetal and newborn growth standards consistent with WHO growth curves and are derived from children in developed and developing countries. However, 22% of infant lengths in our population exceeded InterGrowth-21's maximum standards for length which extend up to 51 cm at a z-score of zero, therefore preventing estimation of gestational age for that group.^19^ In contrast to the InterGrowth-21 curves, which provide growth standards through 42 weeks gestational age, the Fenton growth curves extend through 50 weeks gestational age (length up to 57 cm at a z-score of zero). Nevertheless, if growth standards for InterGrowth-21 curves are expanded, they may be preferred to Fenton growth curves, as they are the current standard used for infants of known gestational age. Another consideration is that Fenton growth curves were designed for assessing postnatal growth of preterm infants, who demonstrate unique growth characteristics postnatally, compared to term infants, particularly with regard to weight gain velocity.^11^ However, the transition incorporated into these curves—from preterm to post-term growth—has been validated and thus it seems reasonable to use them in a mixed population of preterm and term infants.

Fenton growth curves may not fully reflect the anthropometry of infants from LMICs. It may be more appropriate to center length on a z-score other than zero to accurately estimate gestational age in certain LMICs.

Second, despite the application of the multiple case definitions, the dataset for this evaluation is limited by our lack of gestational age estimates, thus making a direct comparison of Fenton growth curves to other case definitions difficult. In order to validate our theory, it will be important to replicate these findings in a population of infants with known and accurate gestational age. Similarly, as several of the case definitions require a known gestational age, our use of estimated gestational age inherently led to a degree of uncertainty that may underestimate or overestimate the number of suspected cases and the background prevalence. These challenges, however, reflect the reality in many LMICs, and, to that end, we believe it is important to explore alternative case definitions that can address potential challenges currently faced by practitioners on the field.

Lastly, infants born before and during the ZIKV epidemic were included in our dataset, which could have influenced the detection of congenital microcephaly. In fact, on regression analysis, we did find a significant association between measured OFC and date of birth. However, once adjusted for gender and estimated gestational age, both of which are critical to the interpretation of head circumference, we found there was no longer a significant association. Similarly, we found no associations between identified microcephaly or head circumference z-score when examining birthdate, considering onset of regional ZIKV transmission, and accounting for time for gestation. Therefore, we conclude that it is unlikely that the onset of local ZIKV transmission significantly impacts our results.

## CONCLUSION

It is important to consider how our understanding and the case definition of microcephaly has evolved during the ZIKV epidemic and what effect changing knowledge of congenital ZIKV infection has on the development of screening programs in LMICs. As the case definitions to date have not fully addressed the limitations of evaluating children of unknown gestational age, the definitions should continue to be reviewed and adjusted as we better understand the clinical presentation of congenital ZIKV infection. However, the research on the causality and long-term implications of microcephaly in developing countries should be prioritized. These are children living in the most resource-constrained settings, with limited access to health care, but having the highest risk factors for exposure to mosquito-borne illnesses. Hence, part of the legacy from this ZIKV epidemic to the global community will be to highlight the need to develop more robust and clear guidelines for identifying which infants require further evaluation.

As the case definitions to date have not fully addressed the limitations of evaluating children of unknown gestational age, the definitions should continue to be reviewed and adjusted as we better understand the clinical presentation of congenital ZIKV infection.

## Notes

**Peer Reviewed**

**Funding:** Jose Fernando Bolanos Menendez Foundation and the Center for Global Health at the University of Colorado.
